# Supplementary material for: An improved digestion and analysis procedure for silicon in plant tissue
Source: PLoS One. 2023 Sep 8;18(9):e0289151. doi: 10.1371/journal.pone.0289151 (PMC10490927; doi:10.1371/journal.pone.0289151)
Supplement: S1 File — (PDF) [file pone.0289151.s001.pdf]

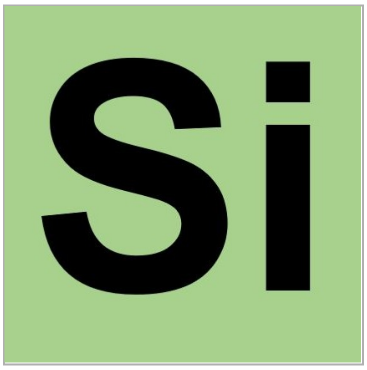

Protocol Info: Noah Langenfeld, Bruce Bugbee . An improved digestion and analysis procedure for silicon in plant tissue. **protocols.io** https://protocols.io/view/an-improved-digestion-and-analysis-procedure-for-s-cngvvbw6

Created: Jan 28, 2023

Last Modified: Apr 06, 2023

PROTOCOL integer ID: 76021

An improved digestion and analysis procedure for silicon in plant tissue

Noah Langenfeld[1], Bruce Bugbee[1]

[1]Utah State University

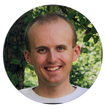

Noah Langenfeld
Utah State University

ABSTRACT

Silicon (Si) in plant tissues reduces abiotic and biotic stress, but it is incorporated as silica (SiO_2), which is difficult to solubilize for analysis. We modified an oven-induced tissue-digestion method to improve Si solubilization and validated the accuracy by quantifying the mass-balance recovery of Si from hydroponic solution and in plant tissues. Leaf, stem, and root tissues were dried, finely-ground, and digested in 12.5 molar sodium hydroxide at 95 °C. The solutions were then acidified with hydrochloric acid to achieve a pH of 2 for measurement of Si using the molybdate blue colorimetric method. Interference of phosphorus (P) in the analysis was minimized by increasing the addition of oxalic acid from 0.6 to 1.1 molar. We recovered 101 ± 13% of the Si in leaf, stem, and root tissues across 15 digestions. This Si recovery was fourteen-fold higher than the standard acid-extraction method and similar to a USDA-ARS alkaline-extraction method. Our procedure offers a low-cost, accurate method for extraction and analysis of Si in plant tissues.

MATERIALS

Sample digestion

- Octyl alcohol
- 100 mM sodium hydroxide
- 30% hydrogen peroxide
- 12.5 M sodium hydroxide
- 5 mM ammonium fluoride
- 6 M hydrochloric acid
- Deionized water

Sample analysis

- Deionized water
- 6 M hydrochloric acid
- 81 mM ammonium molybdate
- 1.1 M oxalic acid
- 90% sucrose and 10% isoascorbic acid

SAFETY WARNINGS

This procedure utilizes a strong base (12.5 M sodium hydroxide) and a strong acid (6 M hydrochloric acid) for sample digestion and fixing. Gloves and safety goggles are **required** when handling these chemicals.

BEFORE START INSTRUCTIONS

Preheat the oven to 95 °C.

Sample drying

1 Dry fresh plant tissue at 80 °C for at least 48:00:00. Water can remain in tissue below 80 °C, which increases dry mass, and volatile compounds can be driven off above 80 °C, which reduces dry mass. 2d

Sample grinding

2 Grind dry plant tissue in a mortar and pestle to a uniform, fine powder. Particle sizes should be less than about 0.1 mm in diameter (consistency of fine sand).

Sample preparation

10m

3 Preheat an oven to 95 °C.

4 Triple rinse a 50-mL polyethylene screw-cap centrifuge tube with [M] 100 millimolar (mM) sodium hydroxide.

5 Triple rinse the 50-mL polyethylene screw-cap centrifuge tube with distilled water.

6 Dry the tube and cap with a clean paper towel.

7 Add about 100 mg of dry and ground plant tissue to the tube. Record the exact mass. Ensure all ground tissue is transferred to the bottom of the tube and not stuck on the side.

First digestion

30m

8 Add 5 drops of octyl-alcohol to the ground tissue in the bottom of the tube to reduce foaming.

9 Add 2 mL of [M] 30 % (v/v) hydrogen peroxide to the bottom of the tube. Wash the inside of the tube free from the tissue sample with the hydrogen peroxide as it is added.

10 Tighten the screw cap and place the tube upright (standing inside a 250 mL glass beaker works well) into a 95 °C oven for 00:30:00 . 30m

Second digestion

4h

11 After 00:30:00 , remove the tube from the oven using heat-safe gloves.

12 Inside a fume hood, add 4 mL of [M] 12.5 Molarity (m) sodium hydroxide to the tube. Add the sodium hydroxide slowly to avoid excess foaming.

13 Gently vortex the tube, replace the cap, and return to the 95 °C oven for an additional 04:00:00 . 4h

Sample fixing

5m

14 After 4 hours, remove the tube from the oven using heat-safe gloves.

15 Add 1 mL of 5 mM ammonium fluoride to the tube to facilitate the formation of monosilicic acid.

16 Add 9 mL of [M] 6 Molarity (m) hydrochloric acid to neutralize the sample. Add the hydrochloric acid slowly to avoid foaming. The solution should turn clear after addition of the acid.

17 Add distilled water to the tube up to 50 mL.

Sample analysis

12m

18 Use deionized water to prepare a 1:25 dilution of the sample with a final volume of 10 mL. Place sample into a 10 mL glass vial or test tube.

19 Add 6 drops of [M] 6 Molarity (m) hydrochloric acid to the sample vial. Cap the vial and invert to mix.

20 Add 12 drops of [M] 81 millimolar (mM) ammonium molybdate. Cap the vial and invert to mix. Wait 00:05:00. 5m

21 Add 8 drops of [M] 1.1 Molarity (m) oxalic acid. Cap the vial and invert to mix. Wait 00:02:00. 2m

22 Add 100 mg of [M] 90 Mass Percent sucrose and [M] 10 Mass Percent isoascorbic acid. Cap the vial and invert to mix until solids have dissolved. Wait 00:05:00 . 5m

23 If using a LaMotte Smart3 colorimeter, select the **Silica - Low Range** method. Insert vial into colorimeter to obtain measurement of silica in the sample. If using a spectrophotometer, prepare a calibration curve from 0 to 4 ppm silica and analyze all samples at 650 and 815 nm.
