# Supplementary material for: An improved digestion and analysis procedure for silicon in plant tissue
Source: PLoS One. 2023 Sep 8;18(9):e0289151. doi: 10.1371/journal.pone.0289151 (PMC10490927; doi:10.1371/journal.pone.0289151)
Supplement: Attachment — Submitted filename: Response to reviewers Si test.docx [file pone.0289151.s002.docx]

Dear PLOS ONE Editor and Reviewers,

We sincerely appreciate the time you took to review our article. Peer reviews are essential to improving our science. Please see below for a point-by-point response to the questions raised during the review process.

Editor

1. Our manuscript meets PLOS ONE’s style requirements to the best of our knowledge.
2. The title on the online submission form and the title in the manuscript do match. The title is “An improved digestion and analysis procedure for silicon in plant tissue”. Line 2 of the manuscript is a short title, as indicated by the PLOS ONE website (<https://journals.plos.org/plosone/s/submission-guidelines#:~:text=Include%20a%20full%20title%20and%20a%20short%20title%20for%20the%20manuscript>)
3. We already completed and uploaded a PDF copy of the protocols.io version. This was included as S1_file.pdf in the original submission. The DOI was generated and was already included in the submission manuscript in both the Associated Content and Materials and Methods Section.

Reviewer 1

1. We focused on cucumber in this manuscript because it is a Si-accumulating species that is also commonly grown in controlled environments. The forms of silica in plant tissue are not species dependent, so we have no reason to believe that these results would differ among other species. The original paper this work is based on, for example, tested both rice and sugarcane, and found a high Si recovery in both species.
2. This method can be conducted with one sample or dozens of samples. As with most analytical tests, time efficiency increases with increasing sample size. This method is therefore no different in its time efficiency than comparative methods. While there are longer heating times, researchers do not have to be present during these times for monitoring, which could potentially increase time efficiency.

Reviewer 2

Line 33: We modified the first sentence of the introduction to say, “Si is the second largest component in the Earth’s crust”. We removed any reference to oxygen, and thus removed confusion.

Line 38: Excellent comment. We have revised the manuscript to maintain consistency of referencing silica vs SiO_2_. Silica is chemically specified as SiO_2_ upon first mention in the manuscript, and it is referred to as silica in subsequent mentions.

Line 39-40: Thank you for this comment. We have added a reference concerning the forms and locations of Si in plants.

Line 57-59: Thank you for this suggestion. We have rewritten this paragraph and have merged it with the subsequent paragraph.

Line 64: We have added a reference to this sentence that provides average concentrations for phosphorus in plant tissue.

Line 66: We have added a reference on the standard colorimetric methods.

Line 71-73: We have rewritten the sentence to aid in clarity.

Line 77-79: This paragraph represents a separate idea from the previous paragraph. So, while short, we felt it has merit to stand on its own.

Line 90-91: We have edited this sentence for clarity.

Line 109-110: Thank you for your question. We have rewritten this paragraph to clarify how the Si was added over the study. Following the principles of mass balance cited earlier in the manuscript, we refill with a dilute nutrient solution throughout the study as needed and accurately quantify how much Si was added to the root-zone over the study.

Line 120-121: This data is shown in table 2. We have rewritten the sentence to specify this.

Line 135: We agree this needed to be clarified. We added a phrase to the sentence to specify how the number 0.467 was derived.

Line 150-152: We do not have a solid reason why solutions become less stable over time. We thus rewrote this section to specify this issue.

Thank you!

Noah J. Langenfeld and Bruce Bugbee

Crop Physiology Laboratory – Utah State University
