# Supplementary material for: An improved digestion and analysis procedure for silicon in plant tissue
Source: PLoS One. 2023 Sep 8;18(9):e0289151. doi: 10.1371/journal.pone.0289151 (PMC10490927; doi:10.1371/journal.pone.0289151)
Supplement: Attachment — Submitted filename: Response to reviewers Si test 2.docx [file pone.0289151.s003.docx]

Dear PLOS ONE Editor and Reviewers,

We sincerely appreciate the time you took to review our article for the second time. We have polished the manuscript to make it clearer and more concise, and we have specifically modified the manuscript to address each of the two comments.

Reviewer 1

1. Thank you for your comments about including the species name in the title. We did an additional literature review to determine the form of Si that is stored in plant tissue. Two manuscripts have found that Si is always stored as silica regardless of species. We reorganized paragraphs to the manuscript and cited these references:

“Plants take up Si as monosilicic acid (Si(OH)_4_) [2], and store it in the same way, as silica (SiO_2_) in leaf cuticles, cellular lumens, and cell walls [7,8].”

[2] Luyckx M, Hausman J-F, Lutts S, Guerriero G. Silicon and plants: Current knowledge and technological perspectives. Front Plant Sci. 2017;8. doi:10.3389/fpls.2017.00411

[7] Sangster AG, Hodson MJ, Tubb HJ. Silicon deposition in higher plants. Studies in Plant Science. Elsevier; 2001. pp. 85–113. doi:10.1016/S0928-3420(01)80009-4

[8] Lanning FC, Ponnaiya BWX, Crumpton CF. The Chemical Nature of Silica in Plants. Plant Physiol. 1958;33: 339–343. doi:10.1104/pp.33.5.339

Since the storage form is uniform among species, extraction should be the same among species. We would like to keep the word ‘cucumber’ out of the title as we feel including it would limit the article impact. The abstract does clearly indicate that the work was done on cucumber because it has high concentration of Si in its tissues.

2. Thank you for your concern about the test length efficiency. We added a paragraph indicating the time it took to run a single sample and multiple samples:

“There is a significant economy of scale in this procedure. A single sample took 5 hours to analyze, while nine samples took 5.25 hours.”

Reviewer 2

1. Thank you for recommending our manuscript for publication!

Thank you!

Noah J. Langenfeld and Bruce Bugbee

Crop Physiology Laboratory – Utah State University
